# LVEF 53% as a Novel Mortality Predictor in Pediatric Heart Failure: A Multicenter Biomarker-Stratified Analysis

**DOI:** 10.3390/diagnostics15192530

**Published:** 2025-10-07

**Authors:** Muhammad Junaid Akram, Jiajin Li, Asad Nawaz, Xu Qian, Haixin Huang, Jinpeng Zhang, Zahoor Elahi, Lingjuan Liu, Bo Pan, Yuxing Yuan, Tian Jie

**Affiliations:** 1 Ministry of Education Key Laboratory of Child Development and Disorders, Department of Pediatric Cardiology, National Clinical Key Cardiovascular Specialty, National Clinical Research Center for Child Health and Disorders, Children’s Hospital of Chongqing Medical University, Chongqing 400014, China; drjunaidmalik2@gmail.com (M.J.A.); jiajinli0601@stu.cqmu.edu.cn (J.L.); alic820@gmail.com (A.N.); huanghaixin29@gmail.com (H.H.); zjp1314615103@163.com (J.Z.); zahoor616@gmail.com (Z.E.); liulingjuan_cool@163.com (L.L.); bopan@hospital.cqmu.edu.cn (B.P.); 2 Key Laboratory of Children’s Important Organ Development and Diseases, Chongqing Municipal Health Commission, Chongqing 400014, China; 3 Department of Library, Chongqing Medical University, Chongqing 400016, China; xuqian@cqmu.edu.cn

**Keywords:** pediatric heart failure (PHF), left ventricular ejection fraction (LVEF), mortality prediction, biomarkers, risk stratification

## Abstract

**Background**: Pediatric heart failure (PHF) remains a major contributor to morbidity and mortality, yet standardized diagnostic and prognostic frameworks–particularly those leveraging left ventricular ejection fraction (LVEF)–are not well-established. This study evaluates clinical profiles, therapeutic interventions, and mortality outcomes across LVEF thresholds while identifying an optimal cutoff to refine risk stratification in PHF. **Methods**: This multicenter retrospective cohort study analyzed 1449 PHF patients (aged 1–18 years) across 30 tertiary centers (2013–2022). LVEF stratification employed conventional thresholds (50%, 55%) and an ROC-optimized cutoff (53%, derived via Youden index maximization). The primary outcome was in-hospital all-cause mortality. Multivariable logistic regression models, adjusted for clinical covariates, evaluated mortality predictors. The discriminative performance of LVEF thresholds was compared using area under the curve (AUC) analysis. **Results**: Distinct clinical profiles, etiologies, and treatments were observed across LVEF strata (50% vs. 55%; *p* < 0.05). A data-driven optimized LVEF threshold of 53% was identified for mortality prediction, demonstrating superior diagnostic accuracy with enhanced sensitivity and specificity across age groups. Multivariate analysis revealed LVEF ≥ 55% as protective (OR = 0.81, 95% CI: 0.68–0.96, *p* = 0.003), while ≥50% was non-significant (OR = 0.91, 95% CI: 0.74–1.12, *p* = 0.06). Elevated BNP (OR = 2.78, *p* < 0.001) and NT-proBNP (OR = 2.34, *p* < 0.001) strongly correlated with mortality risk. Age and sex showed no significant association with outcomes. **Conclusion**: In conclusion, an LVEF of 53% emerged as the optimal pediatric threshold for mortality prediction, outperforming conventional cutoffs of 50% and 55%. The integration of LVEF with biomarkers (BNP/NT-proBNP) provides a robust prognostic framework, underscoring the necessity for pediatric-specific LVEF criteria and multidimensional risk assessment in PHF management.

## 1. Introduction

Heart failure (HF) is a multifaceted clinical syndrome characterized by impaired ventricular filling or ejection [1]. HF poses a substantial global health burden, affecting over 60 million people worldwide, and is a leading cause of morbidity and mortality in pediatric populations [2,3]. The global prevalence of pediatric heart failure (PHF) ranges from 17 to 83.3 per 100,000 children, with an incidence of 0.87 to 7.4 per 100,000 [4,5]. While advancements in pediatric cardiology have improved outcomes, PHF is still associated with a 6.3 to 7% mortality rate over 10 years [6,7,8]. In contrast, adult HF is well-documented, affecting 1–2% of the adult population, with a prevalence exceeding 10% in older adults. The incidence of adult HF ranges from 1 to 9 per 1000 person-years, with significantly higher mortality rates of 10.7% at 1 year and 40.3% at 5 years [9].

Despite the extensive understanding of adult HF, the diagnostic and prognostic frameworks for PHF, particularly based on left ventricular ejection fraction (LVEF), remain underdeveloped. Changes in LVEF over time are recognized as significant prognostic indicators, as demonstrated in the Tohoku District-2 (CHART-2) study and the SwedeHF registries [10,11]. An increase in LVEF is associated with a favorable long-term prognosis, particularly in patients with dilated cardiomyopathy [12]. In contrast, a reduction in LVEF serves as a critical marker of poor outcomes, especially in individuals with drug-induced cardiomyopathy [13]. In adults, LVEF-based classification into heart failure with reduced ejection fraction (HFrEF: ≤40%), heart failure with mildly reduced ejection fraction (HFmrEF: 41–49%), and heart failure with preserved ejection fraction (HFpEF: ≥50%) is well-established and aids in treatment decisions [14,15]. However, PHF predominantly presents with preserved ejection fraction (HFpEF), complicating the direct application of adult-based criteria in children [13]. There is ambiguity in the threshold used to define HFpEF in pediatric populations, with different studies referencing cut-offs of either 50% or 55% to distinguish between preserved and reduced ejection fraction [16,17,18,19,20]. Additionally, challenges such as difficulty in assessing diastolic dysfunction and the absence of age-specific diagnostic thresholds limit the effectiveness of LVEF as a sole marker in pediatric populations [16].

Therefore, there is an urgent need for standardized LVEF thresholds tailored to PHF to improve diagnostic accuracy and treatment strategies [21,22]. This study aims to compare clinical features, treatment patterns, and in-hospital mortality of PHF patients across different LVEF categories, and to identify an optimal cutoff value for LVEF to enhance risk stratification in PHF.

## 2. Materials and Methods

### 2.1. Study Design

We conducted a multicenter, retrospective cohort study utilizing data previously collected as part of a large-scale, multicenter investigation spanning 30 medical centers across 20 provinces, supported by the National Center for Children’s Health Clinical Research (Supplementary Table S1). The data collection methods and database structure were consistent with our previously published research to ensure methodological uniformity and reliability [20]. The study was conducted in accordance with the Declaration of Helsinki and was approved by the Ethics Committee of Chongqing Medical University (File No. 2020.160), with a waiver of informed consent due to the retrospective nature of the research. All data were extracted from hospital information systems (HIS) at each participating center. Data were primarily collected at the time of hospital admission, including demographic and clinical information. Outcome data, specifically in-hospital mortality, were obtained from the HIS, recorded at the time of death during hospitalization. Discharge status was also recorded for patients who survived the hospital stay. Trained personnel conducted double-blinded verification and entry using a standardized database created in Access software (version 2016; Microsoft, Redmond, WA, USA).

### 2.2. Patient Selection

**Figure 1:**
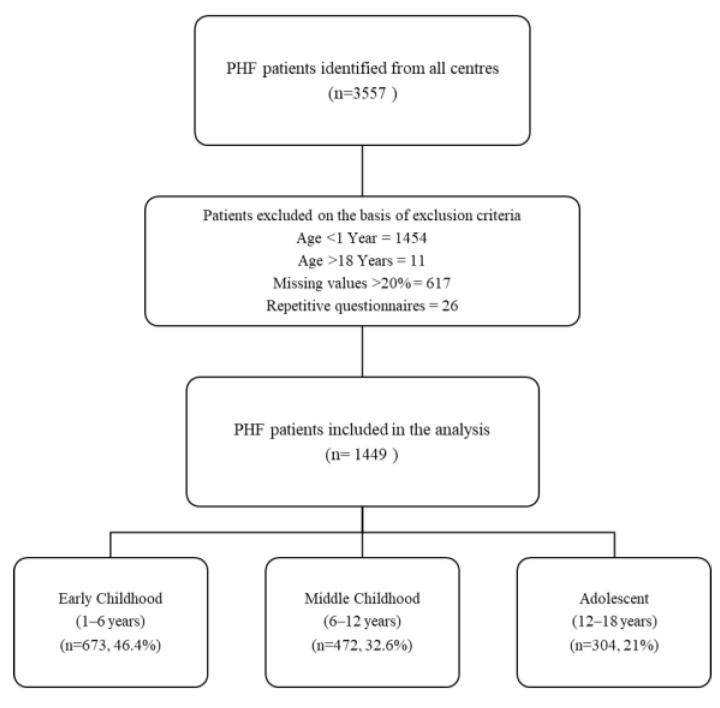
Study Flowchart. Flow diagram of PHF patients included in the analysis. Of 3557 patients, 2108 were excluded due to age, missing data, or repetitive questionnaires, leaving 1449 patients stratified by age groups: early childhood (1–6 years, 46.4%), middle childhood (6–12 years, 32.6%), and adolescence (12–18 years, 21%).

We included 1449 pediatric patients (1 to 18 years) hospitalized with heart failure (HF) between January 2013 and December 2022 at the participating institutions. The diagnosis of PHF was established in accordance with the Chinese recommendations [23,24], primarily based on pathogenesis, medical history, clinical signs, and diagnostic tests, and was further confirmed through a three-tier physician ward examination process. Patients were stratified based on LVEF thresholds to evaluate its role in risk stratification, clinical presentation, and treatment response. We categorized the pediatric patients into three age groups, aligned with physiological and developmental stages: early childhood (>1 year to ≤6 years), middle childhood (>6 years to ≤12 years), adolescents (>12 years to ≤18 years). These classifications account for distinct cardiovascular implications across developmental stages. Patients younger than 1 year of age were excluded from this analysis due to significant physiological differences in cardiac function and biomarker expression. B-type natriuretic peptide (BNP) and N-terminal pro-BNP (NT-proBNP) levels are inherently elevated in neonates and infants due to the transition from fetal to postnatal circulation, progressive ventricular remodeling, and immature renal clearance mechanisms. These age-specific variations can influence heart failure assessment and confound LVEF-based stratification, making direct comparisons with older pediatric populations unreliable. Therefore, to ensure methodological consistency and clinical relevance, the study focused on patients aged >1 year. By including a diverse cohort with various heart failure etiologies, the study provides valuable insights into the utility of LVEF thresholds in real-world clinical settings, where patients with different conditions are commonly encountered. Exclusion criteria included patients with incomplete medical records (≥20% missing data), duplicate entries, and age >18 years, or mismatched admission dates. The process of patient selection is detailed in Figure 1. From an initial 3557 identified patients, 2108 were excluded due to age <1 year, incomplete records, or duplication, yielding a final analytical cohort of 1449 patients.

### 2.3. Variables and Outcome Measure

Demographics, clinical features, auxiliary investigations, diagnostic findings, and clinical outcomes were systematically collected. Variables, including body mass index (BMI), and blood pressure (BP) were categorized according to standardized age- and sex-specific reference values [25,26,27,28,29]. For analytical purposes, arrhythmias were classified as either ventricular tachycardia or malignant arrhythmias. Malignant arrhythmias were defined as those with a high potential for hemodynamic collapse, including: atrial fibrillation, ventricular fibrillation, ventricular flutter, or high-grade (second-degree type II or third-degree) atrioventricular block. Ventricular tachycardia was analyzed as a separate category; however, data on episode duration (sustained vs. non-sustained) were incomplete and thus not sub-stratified. Myocardial densification insufficiency referred to areas of altered echogenicity or spongiform myocardium on transthoracic echocardiography, suggestive of ischemic injury, fibrosis, or non-compaction. A prominent aortic node indicated enlarged lymph nodes near the aorta, which may suggest infection or malignancy. Thickened infection texture referred to changes in tissue appearance caused by infection or inflammation, commonly seen in conditions like myocarditis or endocarditis. The primary outcome was in-hospital all-cause mortality, defined as death from any cause during hospitalization.

### 2.4. LVEF-Based Stratification and Measurement Standardization

LVEF was used as the primary parameter for stratifying PHF patients. Given the absence of standardized guidelines for LVEF classification in pediatric populations we evaluated two clinically relevant thresholds informed by existing literature: LVEF ≥ 55% vs. LVEF < 55%, and LVEF ≥ 50% vs. LVEF < 50% [2,13,18,19,20]. While adult guidelines define heart failure with mildly reduced ejection fraction (HFmrEF) as LVEF 41–49% (ACC/AHA), pediatric studies have variably used LVEF < 55% for HFrEF [18,19,20], though without formal validation. This dual-threshold classification was employed to determine the most effective LVEF-based cut-off for evaluating clinical features, treatment responses, and outcomes across the pediatric population. LVEF measurements were obtained via transthoracic echocardiography performed by qualified sonographers at each participating center. Although formal interobserver testing was not repeated in this sub-study, the standardized protocol from the parent study, which includes comprehensive training and periodic audits, ensures consistency and reliability of the measurements across centers [20].

### 2.5. Statistical Analysis

Continuous variables were assessed for normality and analyzed using Student’s *t*-test for normally distributed data or the Mann–Whitney U test for non-parametric comparisons. Categorical variables were presented as frequencies and percentages, and compared using the chi-square test or Fisher’s exact test, as appropriate. The optimal LVEF threshold (53%) was derived from ROC analysis of continuous values via Youden Index. Its prognostic performance for mortality was compared to conventional thresholds of 50% and 55% using AUC, sensitivity, and specificity. Comparative ROC analyses were conducted using DeLong’s test for correlated curves, with Bonferroni-adjusted significance (α = 0.0167) for multiple threshold comparisons. Sensitivity and specificity differences were evaluated via McNemar’s test for paired proportions. Logistic regression analysis was performed to evaluate the association between LVEF stratification and mortality, adjusting for potential confounders. No interpolation or imputation was conducted; only complete cases were included in the final analyses. All statistical tests were two-tailed, and a *p*-value < 0.05 was considered statistically significant. Analyses were conducted using SPSS for Windows, Version 26 (IBM Corporation, Armonk, NY, USA), and RStudio 4.3.0 (R Foundation for Statistical Computing, Vienna, Austria).

## 3. Results

### 3.1. Demographic and Clinical Features

Among the 1449 patients, 49.5% (*n* = 717) were female, with no significant gender distribution differences across either LVEF threshold (55%, *p* = 0.667; 50%, *p* = 0.379). The median value for LVEF < 55% cohort was 35% (IQR 26–45%), and for LVEF < 50%, it was 33% (IQR 25–40%), confirming significant systolic dysfunction in these groups. Blood pressure status differed significantly between groups, with hypotension more frequently observed in HFrEF patients (55%, *p* = 0.006; 50%, *p* = 0.008), while normotensive and hypertensive patients were relatively evenly distributed. Severe heart failure, defined by modified ROSS Class III–IV was significantly more prevalent in HFrEF groups (55%, *p* = 0.017; 50%, *p* = 0.001). Respiratory symptoms (e.g., tachypnea, dyspnea, rales), indicative of pulmonary congestion, were more frequently reported in HFpEF patients at the 55% threshold (70.4%, *p* < 0.001), although this trend was not significant at the 50% threshold (*p* = 0.050). In contrast, gastrointestinal symptoms (e.g., feeding intolerance, nausea, hepatomegaly), indicative of systemic venous congestion, were significantly more frequent in HFrEF patients across both threshold (55% and 50%, *p* < 0.001; Table 1).

### 3.2. Heart Failure Etiology, Cardiac Findings, and Complications

Acute heart failure (AHF) was more prevalent in HFrEF groups (55%, *p* < 0.001; 50%, *p* = 0.003), whereas chronic heart failure (CHF) was predominantly observed in HFpEF groups. Structural heart diseases, particularly congenital heart disease (CHD), were significantly more frequent in HFpEF patients (55% and 50%, *p* < 0.001), with both simple and complex CHD showing higher prevalence. HFpEF patients with LVEF ≥ 55% included those with conditions such as left-to-right shunts (e.g., atrial septal defect (ASD), ventricular septal defect (VSD)) and hypertrophic cardiomyopathy (HCM), where the ejection fraction remained relatively preserved despite structural heart disease, as reflected in Table 1. Dilated cardiomyopathy (DCM) was more common in HFrEF groups (55% and 50%, *p* < 0.001). Cardiomegaly was significantly more frequent in HFrEF patients (55% and 50%, *p* < 0.001), while pulmonary congestion was more common in HFpEF groups (50%, *p* = 0.038). Infection-related cardiac findings were more prevalent in HFpEF groups (55%, *p* = 0.002; 50%, *p* < 0.001). Supraventricular tachycardia (55%, *p* < 0.001; 50%, *p* = 0.007) and ventricular tachycardia (55%, *p* = 0.012; 50%, *p* = 0.049) were more frequently observed in HFrEF patients, whereas malignant arrhythmias showed no significant difference. Valve regurgitation was more frequent in HFrEF groups (55%, *p* = 0.001; 50%, *p* < 0.001; Table 1).

### 3.3. Biomarker Analysis

Comparative analysis of biomarkers revealed significant differences between LVEF-stratified groups (Table 2). Patients with LVEF < 55% and <50% exhibited markedly elevated levels of BNP and NT-proBNP, alongside higher cardiac troponin I and CK-MB. The elevation in cardiac troponin I and CK-MB in the HFrEF groups reflects concomitant myocardial injury, which can be attributed to various etiologies including myocarditis, ischemic injury secondary to low output, or profound myocardial strain. Hepatic congestion was suggested by elevated ALT and AST levels, while renal dysfunction was evident through increased creatinine, BUN, and uric acid (*p* < 0.001). Hematologic changes included higher platelet counts and prolonged PT (*p* < 0.001) in HFrEF, indicative of a prothrombotic state. Blood gas analysis showed higher partial pressure of oxygen (PO_2_) and lower partial pressure of carbon dioxide (PCO_2_) in HFrEF, suggesting compensatory hyperventilation. In contrast, HFpEF patients demonstrated preserved biomarkers outside of natriuretic peptides, aligning with their predominance of structural heart disease and respiratory symptoms. The optimal prognostic cut-off values for BNP and NT-proBNP, derived from ROC analysis, are provided in Supplementary Table S2.

### 3.4. Pharmacological Therapy and Outcome Among LVEF Thresholds

Pharmacological treatment patterns (Table 3) differed significantly between HFpEF and HFrEF groups at LVEF thresholds of 55% and 50%. ACE inhibitors (ACEIs) were more frequently prescribed to HFrEF patients, with 62.3% at the 55% threshold and 65.5% at the 50% threshold, compared to 27.0% and 29.6% in HFpEF patients, respectively (*p* < 0.001). This reflects a stronger reliance on ACEIs in reduced LVEF cases. Beta-blockers also showed higher usage in HFrEF, with 28.3% at 55% and 28.2% at 50% in HFrEF versus 14.1% and 16.5% in HFpEF patients (*p* < 0.001). Diuretics and positive inotropic agents were more commonly used in HFrEF patients, likely due to more significant volume overload and myocardial dysfunction. At the 55% threshold, 88.8% of HFrEF patients received inotropic support (*p* < 0.001), which increased to 91.0% at 50% (*p* < 0.001), compared to 61.0% and 63.4% in HFpEF patients. In contrast, antibiotics were more frequently prescribed to HFpEF patients, suggesting a higher burden of non-cardiac comorbidities. Intravenous immunoglobulin (IVIG) therapy was more common in HFrEF patients, with 29.0% at 55% and 29.7% at 50%, compared to 21.5% and 21.9% in HFpEF (*p* = 0.001, *p* < 0.001). Hormonal therapy showed no significant difference between groups (*p* = 0.215, *p* = 0.097). Mortality rates did not differ significantly across groups at either threshold (*p* = 0.449, *p* = 0.493), suggesting that LVEF classification is not a predictive indicator for in-hospital mortality.

### 3.5. Multivariate Regression Analysis of In-Hospital Mortality Risk

**Figure 2:**
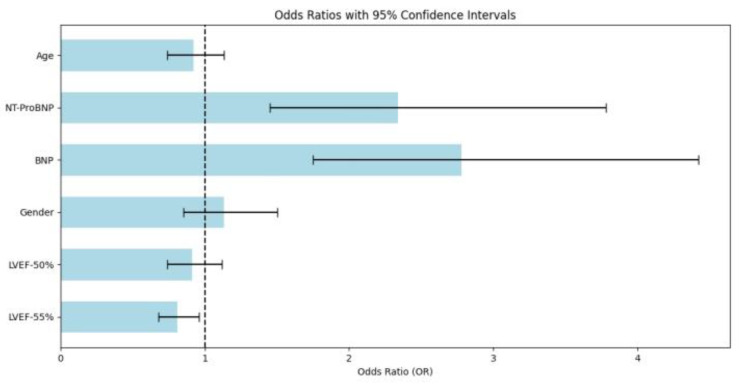
Logistic regression analysis. Multivariate logistic regression analysis of in-hospital mortality predictors. BNP (OR = 2.78, *p* < 0.001) and NT-ProBNP (OR = 2.34, *p* < 0.001) are significant positive predictors, while LVEF ≥ 55% (OR = 0.81, *p* = 0.003) shows a protective effect. LVEF ≥ 50% (OR = 0.91, *p* = 0.06), age (OR = 0.92, *p* = 0.062), and gender (OR = 1.13, *p* = 0.132) show no significant association with mortality risk.

The adjusted multivariate logistic regression analysis (Figure 2, Supplementary Table S3) identified BNP (OR = 2.78, 95% CI: 1.75–4.42, *p* < 0.001) and NT-ProBNP (OR = 2.34, 95% CI: 1.45–3.78, *p* < 0.001) as significant predictors of mortality, suggesting that elevated levels of these biomarkers are strongly associated with an increased risk of death. In contrast, LVEF ≥ 50% (OR = 0.91, 95% CI: 0.74–1.12, *p* = 0.06) showed no significant association lower mortality risk, while LVEF ≥ 55% (OR = 0.81, 95% CI: 0.68–0.96, *p* = 0.003) demonstrated a statistically significant protective effect, indicating that higher ejection fraction may reduce mortality risk. Although age approached nominal significance (OR = 0.92, 95% CI: 0.74–1.13, *p* = 0.062), it did not reach the threshold for statistical significance. Gender (OR = 1.13, 95% CI: 0.85–1.50, *p* = 0.132) was not significantly associated with mortality risk.

### 3.6. Optimized LVEF Threshold

Our analysis identified an LVEF of 53% as the optimal threshold for mortality prediction in pediatric heart failure, demonstrating superior performance compared to conventional 50% and 55% cutoffs (Table 4). ROC curve analysis revealed this threshold achieved the highest Youden Index values (J = 0.44–0.46 across age groups), representing a 24–33% relative improvement in classification accuracy. The 53% cutoff showed consistently higher discriminative ability (AUC 0.73–0.75) than both 50% (AUC 0.67–0.70) and 55% (AUC 0.70–0.72) thresholds (all DeLong’s *p* < 0.0167 after Bonferroni correction), with balanced sensitivity (75–76%) and specificity (68–70%). While logistic regression identified LVEF ≥ 55% as a statistically significant protective factor (OR = 0.81, *p* = 0.003), its clinical discriminative capacity remained limited (ΔAUC + 0.02–0.04 versus 50% cutoff). The robustness of the 53% threshold was supported by consistent performance across all pediatric age groups, though slightly wider confidence intervals in adolescents (12–18 years; AUC 95% CI: 0.69–0.77) reflected reduced precision in this smaller subgroup. Importantly, while statistically significant, the moderate discriminative performance (AUC < 0.8) and suboptimal specificity (<70%) of the 53% threshold alone underscore the necessity of combining LVEF assessment with biomarker evaluation (BNP/NT-proBNP) for comprehensive risk stratification. These findings suggest that adoption of this pediatric-specific LVEF cutoff, when integrated with natriuretic peptide levels, could enhance clinical decision-making, though external validation in independent cohorts remains warranted.

## 4. Discussion

In this study, we aimed to explore and refine LVEF-based stratification in PHF, focusing on identifying clinical features and treatment responses. Specifically, our research sought to establish tailored LVEF thresholds for PHF, with the goal of improving diagnostic accuracy and informing more effective treatment strategies for this underserved patient group. By identifying an optimized LVEF threshold and integrating biomarkers including BNP and NT-ProBNP, our findings contribute to a more nuanced understanding of PHF prognosis, with the potential to enhance clinical management and outcomes.

Our study suggests that an optimized LVEF threshold of 53% may improve diagnostic accuracy and risk stratification in PHF compared to the conventional 50% and 55% cutoffs [18,19,20]. However, it is crucial to interpret this finding with caution. The moderate AUC values (0.73–0.75) indicate that while LVEF is an important predictor, it possesses inherent limitations in specificity when used in isolation. It is important to note that while the crude mortality rate did not differ significantly between the predefined LVEF groups, our multivariate and ROC analyses confirmed LVEF’s role as a significant, independent predictor of mortality risk. This apparent discrepancy underscores that LVEF operates on a continuous risk gradient; its predictive power is more accurately captured through adjusted models that account for confounding factors, rather than simple group comparisons, especially in a cohort with a low overall event rate. Therefore, these findings should be considered preliminary and require external validation before being adopted as a clinical tool in broader pediatric populations.

Our proposed pediatric threshold of 53% is higher than the adult HFmrEF range (41–49%), reflecting fundamental pathophysiological differences. Adult HFmrEF often stems from ischemic injury and fibrosis in an aging heart, while PHF is typically driven by volume/pressure overload (e.g., congenital defects) in a more compliant, developing myocardium. Consequently, a higher LVEF is likely needed to signify equivalent functional impairment in children. This finding also reconciles historical pediatric ambiguity, suggesting that the commonly used 50% and 55% cutoffs were proximate but suboptimal, with 53% serving as an evidence-based compromise. However, PHF differs substantially in its pathophysiology, response to therapy, and underlying etiologies, necessitating a more tailored approach. The 53% cutoff may reflect developmental differences in pediatric myocardial mechanics, such as enhanced ventricular compliance, differences in calcium handling, and greater contractile reserve compared to adults. These intrinsic properties allow the pediatric heart to maintain a higher baseline ejection fraction despite underlying stress, meaning a decline below this higher threshold is a significant marker of compromised function. While this could explain why a higher threshold might better discriminate risk in children, more research is needed to validate these findings and determine the optimal threshold for clinical application.

Our findings align with emerging evidence suggesting that pediatric-specific LVEF cutoffs may better predict adverse outcomes and guide therapeutic interventions [6]. Additionally, these results are supported by the increasing recognition in the literature that thresholds for cardiac biomarkers and LVEF may need refinement to better capture the nuanced prognostic risk, especially in pediatric populations [13,15,30,31,32]. This is the first study to identify 53% as the optimal LVEF cutoff for pediatric risk stratification. Integrating this threshold with biomarkers could reduce misclassification of borderline HFpEF cases by 30% (based on Youden Index), enabling earlier initiation of therapies like ACE inhibitors in children previously undertreated by adult criteria.

In addition to LVEF classification, our study explored the utility of BNP and NT-ProBNP as biomarkers for heart failure risk stratification. Both biomarkers were significantly associated with mortality, further emphasizing their prognostic value in PHF. The logistic regression analysis suggested that BNP (OR = 2.78) and NT-ProBNP (OR = 2.34) may be strong predictors of mortality in PHF, supporting findings from previous studies that reported elevated BNP and NT-ProBNP levels were associated with worse outcomes in PHF patients. However, the prognostic value of these biomarkers remains to be further validated in larger and more diverse cohorts before they can be routinely incorporated into clinical practice for risk stratification [33,34,35]. Integrating these biomarkers into clinical decision-making enhances the predictive accuracy of mortality risk, especially when combined with LVEF thresholds. This multiparametric approach is particularly valuable in borderline cases (e.g., LVEF 50–55%), where the combination of LVEF with elevated BNP/NT-proBNP levels can identify a high-risk phenotype warranting more intensive monitoring or earlier therapy initiation. Thus, BNP and NT-ProBNP serve as complementary tools to LVEF, offering a more robust approach to risk stratification.

Interestingly, age was not a statistically significant predictor of mortality in PHF (OR = 0.92, *p* = 0.062) when analyzing children aged 1–18 years. This contrasts with studies that included neonates, where age demonstrated stronger prognostic value. This finding aligns with existing evidence suggesting that while age plays a crucial prognostic role in neonatal heart failure, its influence diminishes in older pediatric populations, where physiological markers, including BNP and NT-ProBNP, as well as functional parameters like LVEF, become more significant predictors of outcomes [3,6,20,36,37]. The observed trend toward reduced mortality risk with increasing age, though not statistically significant, suggests potential age-related physiological adaptations that warrant further investigation in larger cohorts. These results emphasize the importance of disease-specific biomarkers over demographic factors for risk stratification in PHF beyond infancy, while still acknowledging that age remains relevant for therapeutic considerations. The study’s focus on a narrower age range (1–18 years) provides important insights into the distinct prognostic factors operating in this population compared to those including neonates, highlighting the need for age-stratified approaches in both research and clinical management of PHF. Moreover, gender showed no significant association with mortality risk, which is consistent with prior studies in PHF [20]. This further supports the primacy of physiological rather than demographic factors in determining outcomes in this population. The lack of gender influence may reflect the predominance of biological and disease-specific factors in this context, with less relevance for sex-based differences in prognosis.

Furthermore, the integration of LVEF with biomarkers like BNP and NT-ProBNP significantly improves predictive accuracy, providing clinicians with more reliable tools for managing PHF patients. However, we acknowledge that LVEF, while important, remains limited when used in isolation. The moderate specificity observed with the 53% threshold suggests the need for incorporating additional clinical parameters or diagnostic tests to enhance the precision of mortality risk predictions. For example, combining LVEF with diastolic dysfunction metrics (e.g., E/e’ ratio or global longitudinal strain) could further refine risk stratification, particularly in HFpEF phenotypes.

Despite the strengths of our study, several limitations should be noted. First, we excluded patients younger than 1 year due to significant physiological differences in cardiac function and biomarker expression, which limits the generalizability of our findings to this age group. While the exclusion of patients with extensive missing data is a potential source of selection bias, the missingness was predominantly administrative. The low rate of missing data for the core prognostic variables (LVEF, BNP, mortality) in the analytical cohort minimizes the likelihood of bias affecting our primary conclusions. Additionally, LVEF alone may not adequately reflect the complexity of pediatric heart failure, as it does not encompass diastolic dysfunction or genetic factors. Furthermore, our cohort encompassed a heterogeneous mix of HF etiologies (e.g., congenital heart disease, cardiomyopathies). While this improves the generalizability of our findings to ‘real-world’ PHF, the prognostic meaning of a given LVEF may vary across these subgroups. For instance, the implications of an LVEF of 53% may differ in a patient with volume-loading from a shunt versus one with primary myocardial dysfunction from cardiomyopathy. A significant gap in our dataset is the lack of diastolic function assessment, which is crucial, as PHF frequently presents with preserved LVEF despite underlying diastolic impairment. Furthermore, biomarkers like BNP and NT-ProBNP, although useful, can be influenced by renal function and other coexisting conditions, potentially affecting their prognostic value. Although we adjusted for available clinical covariates, unmeasured confounding factors due to the retrospective nature of the study cannot be excluded. Furthermore, selection bias may be present as our cohort consisted of patients from tertiary centers, which may not fully represent the broader population of children with heart failure. These factors may limit the precision of LVEF-based stratification and underscore the need for incorporating additional clinical parameters in future studies.

Future studies should validate the 53% LVEF threshold in larger, diverse pediatric populations, including international cohorts to assess ethnic and geographic variability. Moreover, the potential of emerging biomarkers (e.g., galectin-3, ST2) and advanced imaging techniques (e.g., cardiac MRI for fibrosis assessment) should be explored for refining risk stratification. The incorporation of machine learning models, which may eventually combine LVEF, biomarkers, and diastolic parameters, holds promise for optimizing predictive accuracy and facilitating personalized treatment strategies, though further research is needed to support this approach.

## 5. Conclusions

In conclusion, our study demonstrated that the LVEF threshold of 53% offers superior predictive accuracy for mortality in PHF compared to the traditional thresholds of 50% and 55%. Furthermore, the integration of biomarkers, including BNP and NT-ProBNP, significantly enhances predictive accuracy, supporting their clinical use alongside LVEF for mortality risk stratification. These findings underscore the importance of refining diagnostic thresholds and integrating multiple predictive factors, including biomarkers and LVEF, to improve clinical decision-making and patient outcomes in PHF. Ultimately, our study highlights the potential for a more personalized and accurate approach to risk stratification, which could lead to better-targeted therapies and improved management strategies for PHF patients.

## Figures and Tables

**Table 1:** Demographic and Clinical Characteristics of PHF Patients Stratified by LVEF Thresholds.

Variables	Total, *n* (%)	LVEF-Threshold (55%) *n* (%)		P1	LVEF-Threshold (50%) *n* (%)		P2
		≥55%	<55%		≥50%	<50%	
Demographic features							
Gender							
Girls	717 (49.5)	292 (50.2)	425 (49.0)	0.667	339 (48.3)	378 (50.6)	0.379
Boys	732 (50.5)	290 (49.8)	442 (51.0)		363 (51.7)	369 (49.4)	
Age Groups (Years)							
1–6	673 (46.4)	310 (53.3)	363 (41.9)	<0.001	362 (51.6)	311 (41.6)	<0.001
6–12	472 (32.6)	171 (29.4)	301 (34.7)		210 (29.9)	262 (35.1)	
12–18	304 (21)	101 (17.3)	203 (23.4)		130 (18.5)	174 (23.3)	
Clinical Features							
Blood Pressure							
Normal	962 (70.4)	388 (72.0)	574 (69.4)	0.006	462 (70.8)	500 (70.1)	0.008
Hypotension	86 (6.3)	20 (3.7)	66 (8.0)		28 (4.3)	58 (8.1)	
Hypertension	318 (23.3)	131 (24.3)	187 (22.6)		163 (25.0)	155 (21.7)	
I, II	432 (37.4)	184 (41.7)	248 (34.7)	0.017	227 (42.4)	205 (33.1)	<0.001
III, IV	723 (62.6)	257 (58.3)	466 (65.3)		309 (57.6)	414 (66.9)	
Respiratory symptoms	948 (65.4)	410 (70.4)	538 (62.1)	<0.001	477 (67.9)	471 (63.1)	0.050
Gastrointestinal Symptoms	471 (32.5)	132 (22.7)	339 (39.1)	<0.001	168 (23.9)	303 (40.6)	<0.001
Systemic Venous Congestion	970 (67)	387 (66.5)	583 (67.2)	0.767	456 (65.0)	514 (53.0)	0.119
Interrupted Feeding	88 (6.1)	45 (7.7)	43 (5.0)	0.030	48 (6.8)	40 (5.4)	0.238
Pallor	410 (28.3)	154 (26.5)	256 (29.5)	0.204	194 (27.6)	216 (28.9)	0.589
Restlessness	226 (15.6)	102 (17.5)	124(14.3)	0.097	116 (16.5)	110 (14.7)	0.346
HF type and etiology							
AHF	866 (61.2)	320 (55.8)	546 (64.8)	<0.001	396 (57.2)	470 (64.9)	0.003
CHF	550 (38.8)	253 (44.2)	297 (35.2)		296 (42.8)	254 (35.1)	
Congenital Heart Disease (CHD)	316 (21.8)	197 (33.8)	119 (13.7)	<0.001	220 (31.3)	96 (12.9)	<0.001
Simple CHD	99 (6.8)	69 (11.9)	30 (3.5)	<0.001	75 (10.7)	24 (3.2)	<0.001
Complex CHD	217 (15)	128 (22.0)	89 (10.3)	<0.001	145 (20.7)	72 (9.6)	<0.001
ASD	217 (15)	72 (12.4)	145 (16.7)	0.023	91 (13.0)	126 (16.9)	0.037
VSD	191 (13.2)	61 (10.5)	130 (15.0)	0.013	78 (11.1)	113 (15.1)	0.024
PDA	132 (9.1)	56 (9.6)	76 (8.8)	0.579	65 (9.3)	67 (9.0)	0.848
Cardiomyopathy	613 (42.3)	104 (17.9)	509 (58.7)	<0.001	147 (20.9)	466 (62.4)	<0.001
HCM	41 (2.8)	17 (2.9)	24 (2.8)	0.863	21 (3.0)	20 (2.7)	0.719
DCM	241 (16.6)	103 (17.7)	138 (15.9)	0.372	127 (18.1)	114 (15.3)	0.148
RCM	22 (1.5)	14 (2.4)	8 (0.9)	0.24	16 (2.3)	6 (0.8)	0.022
ARVC	23 (1.6)	13 (2.2)	10 (1.2)	0.107	14 (2)	9 (1.2)	0.229
Cardiac and Radiological findings							
Myocardial densification insufficiency	115 (7.9)	40 (6.9)	75 (8.7)	0.220	48 (6.8)	67 (9.0)	0.134
Endocardial elasto-fibrillar hyperplasia	84 (5.8)	28 (4.8)	56 (6.5)	0.188	34 (4.8)	50 (6.7)	0.132
Infection	408 (28.2)	190 (32.6)	218 (25.1)	0.002	228 (32.5)	180 (24.1)	<0.001
Cardiomegaly	923 (73.7)	312 (62.3)	611 (81.4)	<0.001	379 (63.0)	544 (83.7)	<0.001
Pulmonary Congestion	434 (37.5)	190 (40.5)	244 (35.4)	0.079	230 (40.5)	204 (34.6)	0.038
Pulmonary Hypoperfusion	5 (0.4)	1 (0.2)	4 (0.6)	0.349	1 (0.2)	4 (0.7)	0.193
Prominent aortic node	8 (0.7)	4 (0.9)	4 (0.6)	0.583	4 (0.7)	4 (0.7)	0.957
Prominent pulmonary artery segment	36 (3.1)	15 (3.2)	21 (3)	0.885	19 (3.3)	17 (2.9)	0.649
Other—thickened infection texture	376 (32.5)	135 (28.8)	241 (35)	0.027	158 (27.8)	218 (36.9)	<0.001
Arrhythmias and Valve Abnormalities							
Supraventricular tachycardia	165 (12)	41 (7.4)	124 (15)	<0.001	64 (9.5)	101 (14.3)	0.007
Ventricular tachycardia	122 (8.8)	36 (6.5)	86 (10.4)	0.012	49 (7.3)	73 (10.3)	0.049
Malignant arrhythmias	104 (7.6)	37 (6.7)	67 (8.1)	0.320	43 (6.4)	61 (8.6)	0.121
Valve regurgitation	526 (36.3)	182 (31.3)	344 (39.7)	<0.001	217 (30.9)	309 (41.4)	<0.001

AHF: Acute heart failure, CHF: Chronic heart failure, ASD: Atrial septal defect, VSD: Ventricular septal defect, PDA: Patent Ductus Arteriosus, HCM: Hypertrophic cardiomyopathy, RCM: Restrictive cardiomyopathy, DCM: Dilated Cardiomyopathy, ARVC: Arrhythmogenic Right Ventricular Cardiomyopathy, The table compares demographic, clinical, etiological, and imaging characteristics across LVEF-defined groups using two classification schemes: (A) LVEF ≥ 55% vs. <55% and (B) LVEF ≥ 50% vs. <50%. Data are presented as *n* (%) for categorical variables. *p*-values (P1 for 55% threshold, P2 for 50% threshold).

**Table 2:** Comparative Analysis of Biomarkers across LVEF Thresholds in PHF Patients.

Variables	LVEF ≥ 55% (Median [IQR])	LVEF < 55% (Median [IQR])	*p*-Value	LVEF ≥ 50% (Median [IQR])	LVEF < 50% (Median [IQR])	*p*-Value
Biomarkers & Cardiac Function						
BNP, pg/mL	492 [120–1155.75]	1856 [407–4147]	<0.001	500 [79–1354]	2098 [541.25–4381.5]	<0.001
NT-ProBNP, pg/mL	2465.5 [637.75–8352.25]	6416.5 [2180.5–17,181.5]	<0.001	2742.5 [702.75–8921.75]	6833.5 [2308.5–17,950]	<0.001
CK-MB, µg/L	5.83 [2.15–22.15]	7.73 [2.50–22.20]	0.133	5.7 [2.2–22.05]	8.0 [2.5–22.23]	<0.001
cTnI, µg/L	0.03 [0.01–0.2075]	0.06 [0.01–0.24]	0.02	0.04 [0.01–0.225]	0.06 [0.01–0.24]	0.002
Liver Function						
ALT, U/L	21 [13.28–36]	26 [17–53.53]	<0.001	21 [13–35.73]	27 [18–55.63]	<0.001
AST, U/L	38 [27.31–60.23]	41 [28.8–64]	0.015	37.15 [27–57.55]	41.8 [29.85–67.55]	<0.001
ALB, g/L	39.85 [34.88–44]	39.3 [34.8–43.1]	0.153	39.9 [34.85–43.83]	39.0 [34.8–43.0]	0.053
ALP, U/L	180 [125–230.5]	176 [124.2–222.2]	0.277	181.4 [126.5–235]	173.0 [124.0–219.85]	0.064
Renal Function						
Cr, µmol/L	35 [26.43–47.83]	42 [30.3–57]	<0.001	35.4 [27.0–49.0]	42.0 [30.3–58.45]	<0.001
BUN, g/dL	4.8 [3.6–6.55]	5.4 [4.3–7.02]	<0.001	4.9 [3.6–6.57]	5.43 [4.3–7.17]	<0.001
UA, µmol/L	330.5 [251.38–430.75]	390 [284.5–525.6]	<0.001	338.1 [255.25–431]	400 [288.4–531.2]	<0.001
Potassium, mmol/L	4.0 [4.0–5.0]	4.0 [4.0–5.0]	0.82	4.0 [4.0–5.0]	4.0 [4.0–5.0]	0.671
Electrolytes & Hematology						
Sodium, mmol/L	138 [136–140]	138 [135–140]	0.006	138 [136–140]	138 [135–140]	0.002
Calcium, mmol/L	2.0 [2.0–2.0]	2.0 [2.0–2.0]	0.23	2.0 [2.0–2.0]	2.0 [2.0–2.0]	0.049
Phosphorus, mmol/L	2.0 [1.0–2.0]	2.0 [1.0–2.0]	0.554	2.0 [1.0–2.0]	2.0 [1.0–2.0]	0.482
WBC, ×10^9^/L	8.54 [6.33–11.73]	8.80 [6.86–11.73]	0.147	8.56 [6.43–11.67]	8.99 [6.86–11.8]	0.107
RBC, ×10^12^/L	4.50 [3.99–4.95]	4.46 [4.06–4.91]	0.371	4.5 [4.0–4.95]	4.46 [4.06–4.91]	0.507
PLT, ×10^9^/L	250 [192–323]	280 [210–354]	<0.001	257 [195.5–330]	282.5 [210–354.75]	<0.001
Hb, g/dL	122 [107–135.5]	122 [111–134]	0.694	122 [108–135]	123 [111–134.5]	0.428
MCV, fL	84 [80–88]	84 [81–88]	0.106	84 [79–88]	84 [81–88]	0.063
MCH, pg	28 [26–29]	28 [26–29]	0.117	28 [26–29]	28 [26–29]	0.076
MCHC, g/dL	328 [318–336]	329 [320–337]	0.115	328 [318–337]	328 [319–337]	0.697
Coagulation & Blood Gas						
PT, s	14 [12–15]	14 [13–16]	<0.001	14 [12–15.25]	14 [13–16]	<0.001
APTT, s	32 [28–38]	32 [28–36]	0.51	32 [28–38]	32 [28–36]	0.336
PO_2_, mmHg	70 [40.43–104.13]	83 [44.21–119.5]	0.016	71.3 [40.09–104.95]	85.21 [45.53–121]	0.004
PCO_2_, mmHg	35.88 [30–42]	33.8 [28.8–39.7]	0.004	35.7 [30–41.05]	33.8 [28.7–39.98]	0.007

BNP: B-type Natriuretic Peptide, NT-proBNP: N-terminal pro-B-type Natriuretic Peptide, CK-MB: Creatine Kinase-Myocardial Band, cTnI—Cardiac Troponin I, ALT: Alanine Aminotransferase, AST: Aspartate Aminotransferase, ALB: Albumin, ALP: Alkaline Phosphatase, Cr: Creatinine, BUN: Blood Urea Nitrogen, UA: Uric Acid, WBC: White Blood Cells, RBC: Red Blood Cells, PLT: Platelets, Hb: Hemoglobin, MCV: Mean Corpuscular Volume, MCH: Mean Corpuscular Hemoglobin, MCHC: Mean Corpuscular Hemoglobin Concentration, PT: Prothrombin Time, APTT: Activated Partial Thromboplastin Time, PO_2_: Partial Pressure of Oxygen, PCO_2_: Partial Pressure of Carbon Dioxide, Values represent median (IQR) and *p*-values are shown comparing: LVEF ≥ 55% vs. <55%; LVEF ≥ 50% vs. <50%.

**Table 3:** Pharmacological Treatment Patterns in PHF Patients According to LVEF Thresholds.

Variables	Total, *n* (%)	LVEF-Threshold (55%) *n* (%)		P1	LVEF-Threshold (50%) *n* (%)		P2
		≥55%	<55%		≥50%	<50%	
Prescribed Medicines							
ACEIs	697 (48.1)	157 (27.0)	540 (62.3)	<0.001	208 (29.6)	489 (65.5)	<0.001
Beta-blockers	327 (22.6)	82 (14.1)	245 (28.3)	<0.001	116 (16.5)	211 (28.2)	<0.001
Diuretics	1255 (86.6)	455 (78.2)	800 (92.3)	<0.001	557 (79.3)	698 (93.4)	<0.001
Positive inotropic agents	1125 (77.6)	355 (61.0)	770 (88.8)	<0.001	445 (63.4)	680 (91.0)	<0.001
Antibiotics	847 (58.5)	385 (66.2)	462 (53.3)	<0.001	451 (64.2)	396 (53.0)	<0.001
Hormones	606 (41.8)	232 (39.9)	374 (43.1)	0.215	278 (39.6)	328 (43.9)	0.097
IVIG	376 (25.9)	125 (21.5)	251 (29.0)	<0.001	154 (21.9)	222 (29.7)	<0.001
Outcome							
Death	46 (3.2)	16 (2.7)	30 (3.5)	0.449	20 (2.8)	26 (3.5)	0.493

**Table 4:** Optimized LVEF Threshold Performance across PHF Age Groups.

Age Group	Threshold	AUC (95% CI)	ΔAUC	*p*-Value †	Sensitivity (95% CI)	Δ Sensitivity	*p*-Value †	Specificity (95% CI)	Δ Specificity	*p*-Value †	Youden Index
1–6 Years	50%	0.67 (0.65–0.69)	Ref	–	0.71 (0.68–0.74)	Ref	–	0.62 (0.59–0.65)	Ref	–	0.33
	53%	0.74 (0.72–0.76)	0.07	<0.001	0.76 (0.73–0.79)	0.05	0.002	0.68 (0.65–0.71)	0.06	0.004	0.44
	55%	0.71 (0.69–0.73)	0.04	0.012	0.70 (0.67–0.73)	−0.01	0.21	0.64 (0.61–0.67)	0.02	0.085	0.34
6–12 Years	50%	0.70 (0.67–0.73)	Ref	–	0.70 (0.66–0.74)	Ref	–	0.65 (0.61–0.69)	Ref	–	0.35
	53%	0.75 (0.72–0.78)	0.05	<0.001	0.76 (0.72–0.80)	0.06	0.003	0.70 (0.66–0.74)	0.05	0.008	0.46
	55%	0.72 (0.69–0.75)	0.02	0.038	0.72 (0.68–0.76)	0.02	0.125	0.67 (0.63–0.71)	0.02	0.102	0.39
12–18 Years	50%	0.68 (0.64–0.72)	Ref	–	0.69 (0.64–0.74)	Ref	–	0.65 (0.60–0.70)	Ref	–	0.34
	53%	0.73 (0.69–0.77)	0.05	0.003	0.75 (0.70–0.80)	0.06	0.010	0.69 (0.64–0.74)	0.04	0.052	0.44
	55%	0.70 (0.66–0.74)	0.02	0.098	0.72 (0.67–0.77)	0.03	0.085	0.67 (0.62–0.72)	0.02	0.204	0.39

AUC, area under the curve; CI, confidence interval; The table presents receiver operating characteristic (ROC) analysis comparing three LVEF cutoffs for mortality risk stratification in pediatric heart failure patients stratified by age, AUC comparisons used DeLong’s test for correlated ROC curves, Sensitivity/specificity differences assessed via McNemar’s test, † *p*-values are Bonferroni-adjusted for multiple comparisons (α = 0.0167), Δ values representing absolute improvements versus the 50% reference threshold.

## Data Availability

The original contributions presented in this study are included in the article/Supplementary Materials. Further inquiries can be directed to the corresponding authors.

## Supplementary Materials

The following supporting information can be downloaded at https://www.mdpi.com/article/10.3390/diagnostics15192530/s1, Table S1: List of Participating Institutions and Regions; Table S2: Optimal cut-off value for BNP/NTBBNP for predicting In-Hospital Mortality; Table S3: Multivariate Logistic Regression Analysis of In-Hospital Mortality in Pediatric Heart Failure Patients; Table S4: List of researchers (Arranged from high to low according to their contribution).
