# Supplementary material for: LVEF 53% as a Novel Mortality Predictor in Pediatric Heart Failure: A Multicenter Biomarker-Stratified Analysis
Source: Diagnostics (Basel). 2025 Oct 7;15(19):2530. doi: 10.3390/diagnostics15192530 (PMC12524215; doi:10.3390/diagnostics15192530)
Supplement: Supplementary file 1 [file diagnostics-15-02530-s001.zip › diagnostics-3866600-supplementary.pdf]

Supplementary Table S1. List of Participating Institutions and Regions

Province or Municipality	Center
Beijing City	Fuwai hospital，Chinese Academy of Medical Sciences, Peking Union Medical College
	Beijing An Zhen Hospital of the Capital University of Medical Sciences
Tianjin City	Tianjin Children's Hospital
Hebei Province	Hebei Children's Hospital
	The Second Hospital of Hebei Medical University
Inner Mongolia Autonomous Region	Inner Mongolia People's Hospital
Liaoning Province	Shengjing Hospital of China Medical University
Jilin Province	The First Bethune Hospital of Jilin University
Shanghai City	Children's Hospital of Fudan University
	Shanghai Children’s Medical Center, School of Medicine, Shanghai Jiao Tong University
	Xinhua Hospital Affiliated to Shanghai Jiaotong University School of Medicine
Jiangsu Province	Children's Hospital of Nanjing Medical University
Zhejiang Province	Children's Hospital, Zhejiang University School of Medicine
Anhui Province	Anhui Provincial Hospital
Jiangxi Province	Jiangxi Children's Hospital
Shandong Province	Qingdao Women and Children's Hospital
	Children's Hospital Affiliated to Shandong University, Jinan Children's Hospital
	Qilu Hospital of Shandong University
Henan Province	Henan Children's Hospital, Children's Hospital Affiliated to Zhengzhou University
	The First Affiliated Hospital of Zhengzhou University
Hunan Province	Hunan Children's Hospital
Guangdong Province	Guangdong Provincial People's Hospital, Guangdong Academy of Medical Sciences
	Guangzhou Women and Children's Medical Center，Guangzhou Medical University
Chongqing City	Children’s Hospital of Chongqing Medical University
Sichuan Province	Sichuan Academy of Medical Sciences & Sichuan Provincial People's Hospital
	Sichuan Provincial Maternity and Child Health Care Hospital
	The Affiliated Hospital of Southwest Medical University
Tibet Autonomous Region	Tibet Autonomous Region People's Hospital
Shaanxi Province	Xi'an Children's Hospital
Xinjiang Uygur Autonomous Region	The First Affiliated Hospital of Shihezi

The list is presented without any specific order or ranking.

Supplementary Table S2. Optimal cut-off value for BNP/NTBBNP for predicting In-Hospital Mortality in Pediatric Heart Failure Patients

Biomarker	Optimal Cut-off (pg/mL)	AUC	Sensitivity (%)	Specificity (%)	Youden Index
**BNP**	1257	0.64	82.6	45.2	0.278
**NT-proBNP**	5650	0.68	78.1	56.1	0.342

Supplementary Table S3. Multivariate Logistic Regression Analysis of In-Hospital Mortality in Pediatric Heart Failure Patients

Variable	OR (95% CI)	P-Value
LVEF-55%	0.81 (0.68-0.96)	0.003
LVEF-50%	0.91 (0.74-1.12)	0.06
Gender	1.13 (0.85-1.5)	0.132
BNP	2.78 (1.75-4.42)	<0.001
NT-ProBNP	2.34 (1.45-3.78)	<0.001
Age	0.92 (0.74-1.13)	0.062

Supplementary Table S4. List of researchers (Arranged from high to low according to their contribution)

S.No.	Researchers along with their Affiliations
1	Children’s Hospital of Chongqing Medical University (Zhilin Huang, Huichao Sun, Lingjuan Liu, Tiewei Lv);
2	Henan Children's Hospital, Children's Hospital Afliated to Zhengzhou University (Fangjie Wang, Xiaochen Yan, Xiaoli Yao, Yingying Li);
3	Hunan Children's Hospital (Zhi Chen);
4	Shanghai Children’s Medical Center, School of Medicine, Shanghai Jiao Tong University (Ying Guo);
5	The Affiliated Hospital of Southwest Medical University (Xing Shen);
6	Children's Hospital of Fudan University (Fang Liu, Xuecun Liang, Yixiang Lin, Lan Ye);
7	Inner Mongolia People's Hospital (Hua Zhu, Yanyan Liang);
8	Shengjing Hospital of China Medical University (Yanlin Xing, Hong Wang, Wei Liu);
9	Fuwai hospital，Chinese Academy of Medical Sciences, Peking Union Medical College (Huili Zhang);
10	Hebei Children's Hospital (Yingqian Zhang, Bo Li);
11	Children's Hospital, Zhejiang University School of Medicine (Chunhong Xie, Yue Huang, Jianmei Zhu, Zhe Lin);
12	Children's Hospital of Nanjing Medical University (Shiwei Yang, Lianfu Ji);

13	Tianjin Children's Hospital (Shuhua Xing, Jiegang Deng);
14	The First Bethune Hospital of Jilin University (Yanyan Han, Shu Nie, Huaqing Sun);
15	The Second Hospital of Hebei Medical University (Lin Feng, Wei Xu, Haoyun Zhao);
16	The First Affiliated Hospital of Zhengzhou University (Jindou An, Song Feng);
17	Guangzhou Women and Children's Medical Center，Guangzhou Medical University (Li Zhang, Xiaofei Xie);
18	Qilu Hospital of Shandong University (Cuifen Zhao, Minmin Wang, Haizhao Zhao);
19	Qingdao Women and Children's Hospital (Zipu Li, Benzhen Wang, Guangsong Shan);
20	Xi'an Children's Hospital (Juanli Wang, Hongyu Xiao, Huan Li);
21	Tibet Autonomous Region People's Hospital (Bianbazhuoga, Mei Chen, Longya Qiao, Xiangyan Zhong, Yude Ma);
22	Jiangxi Provincial Children's Hospital (Junkai Duan, Fei Xu, Yunguo Zhou, Fang Xu);
23	Sichuan Academy of Medical Sciences & Sichuan Provincial People's Hospital (Qian Peng, Xiaoping Hu, Bo Li);
24	Guangdong Provincial People's Hospital, Guangdong Academy of Medical Sciences (Zhaofeng Xie, Yan Guan, Zhiwei Zhang, Shuishu Wang);
25	Anhui Provincial Hospital (Mei Xiong);
26	Xinhua Hospital Affiliated to Shanghai Jiaotong University School of Medicine (Yurong Wu);
27	Children's Hospital Affiliated to Shandong University, Jinan Children's Hospital (Lei Li, Xinxiang Li);
28	Beijing An Zhen Hospital of the Capital University of Medical Sciences (Yongmei Liang);
29	The First Affiliated Hospital of Shihezi University (Jinyong Pan, Qiang Gu, Fang Jiang);
30	Sichuan Provincial Maternity and Child Health Care Hospital (Xianmin Wang).
